# Diffusion-weighted magnetic resonance spectroscopy with selective refocusing

**DOI:** 10.1007/s10334-025-01275-x

**Published:** 2025-07-15

**Authors:** Emile Berg, Renate Grüner, John Georg Seland

**Affiliations:** 1 Department of Chemistry, University of Bergen, Allegaten 41, 5007 Bergen, Norway; 2 Department of Physics and Technology, University of Bergen, Allegaten 55, 5007 Bergen, Norway

**Keywords:** MRS, Diffusion, Selective refocusing, Spectral editing

## Abstract

**Objective:**

To reduce errors from J-modulations and spectral overlap in dMRS of brain metabolites, this study combines the use of diffusion-weighted gradients with selective refocusing and spectral editing.

**Materials and methods:**

Bipolar gradients were combined with spectral refocusing and editing in a dMEGA-PRESS sequence. Experimental parameters were optimised for spectral editing of GABA, with co-editing of Glutamate and Glutamine. The method was tested in metabolite phantom solutions, followed by pre-clinical experiments on rats.

**Results:**

The dMEGA-PRESS sequence enabled reliable spectral editing and quantification of GABA. Selective refocusing and editing resulted in reduced uncertainty in the diffusion data for GABA and Glutamate in the metabolite phantoms, and also for the combined Glutamate/Glutamine diffusion data obtained in vivo. Reliable diffusion data for GABA was not possible to obtain from the in vivo spectra.

**Discussion:**

For metabolites with significant J-modulations but without spectral overlap, selective refocusing improved the quality of diffusion data. For metabolites with spectral overlap where editing is necessary, spectral subtraction makes it more challenging to improve the quality of diffusion-weighted data.

**Conclusion:**

The dMEGA-PRESS sequence reduces the uncertainty in obtained diffusion data for brain metabolites that are significantly influenced by J-modulations.

**Supplementary Information:**

The online version contains supplementary material available at 10.1007/s10334-025-01275-x.

## Introduction

In biological tissue with differences in physical and chemical environments and geometrical restrictions, molecular displacements of water molecules are described using an apparent diffusion coefficient (ADC). Insight into micro-environmental and biochemical processes in the brain, in particular a mapping of tissue microstructures at the subvoxel level [1], can thus be obtained using diffusion-weighted magnetic resonance imaging (dMRI) [2].

When diffusion-weighting is combined with $^1$H magnetic resonance spectroscopy (dMRS), ADC values of molecules other than water are measured [3–6], potentially providing valuable information about microstructure with a higher specificity compared to dMRI [7]. Principles and methodological considerations for optimised dMRS can be found in review and consensus articles [3, 4, 6, 7]. Reliable ADC values have mainly been obtained for brain metabolites with relatively intense singlet signals, such as N-acetyl aspartate (NAA), choline (Cho), and creatine (Cr) [8]. Other metabolites often suffer from low signal-to-noise ratio, strong spectral overlap, and are often significantly influenced by J-modulations, making dMRS more challenging, in particular at clinical field strengths $(\le 3$ T) [5, 9]. For metabolites with low abundance and strong spectral overlap very few reliable dMRS studies are available.

Consensus and recommendations for dMRS were recently presented [6]. Since diffusion-weighting based on regular spin echo (PRESS) and stimulated echo (STEAM) suffer from B$_1^+$ inhomogeneities and chemical shift displacements, in particular at high magnetic field strengths, methods with adiabatic or semi-adiabatic rf-pulses, respectively LASER or sLASER (Localization by (semi) Adiabatic SElective Refocusing) have been established. In general, DW-STEAM or DW-sLASER are the recommended methods for clinical scanners, while for preclinical scanners with stronger gradients and higher specific absorption rate limits, the selection is larger, enabling use of DW-LASER, but also methods where the diffusion and localisation blocks are separated (SE-LASER or STE-LASER) [6].

The main strategy for low abundant metabolites with potential influence from J-modulations, is to keep the echo time as short as possible, making DW-STEAM preferable on clinical scanners, while STE-LASER is the recommendation on preclinical scanners [6]. Another more recent approach, for preclinical scanners at high magnetic fields, suggests to use DW-SPECIAL (diffusion-weighted-SPin ECho full Intensity Acquired Localized) [10], which incorporate adiabatic pulses but with shorter echo times compared to STE-LASER, reducing the influence from J-modulations. Two-dimensional diffusion-weighted J-resolved methods have also been reported, but this necessitates relatively long scanning times [9, 11].

The study presented in this paper takes advantage of spectral selective refocusing pulses, commonly applied in MRS editing techniques [12], to compensate for strong J-modulations of specific metabolite signals. This is achieved through a diffusion-weighted MEGA-PRESS (dMEGA-PRESS) sequence, where selective refocusing in MEGA-PRESS [13] is combined with bipolar diffusion gradients to achieve high b values [4], and enhance and potentially isolate less intense signals originating from specific metabolites influenced by J-modulations. We demonstrate how diffusion-weighted signals from GABA and glutamate/glutamine can be refocused, enhanced, and isolated using this approach. However, the suggested dMEGA-PRESS method is suitable for any metabolite that benefits from selective refocusing and spectral editing.

Notably, a diffusion-weighted MEGA-LASER method would be more robust with respect to B$_1^+$ inhomogeneities and chemical shift displacements. However, on most pre-clinical scanners PRESS is readily available, while a LASER method would have to be implemented and optimised with respect to adiabatic pulses and time intervals. Therefore, as a ‘proof of principle’ approach PRESS was chosen as a fundamental building block. A diffusion-weighted MEGA-LASER method will however be pursued in future studies.

## Theory

**Fig. 1:**
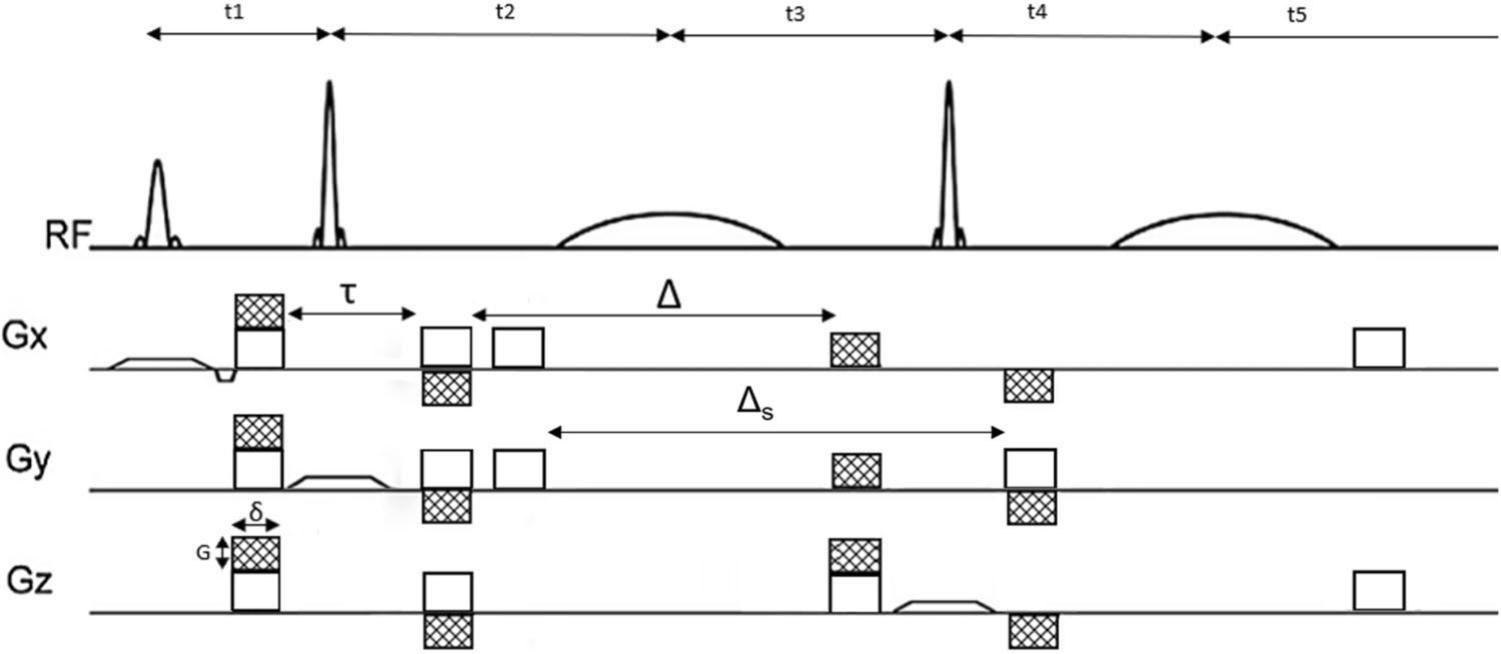
The dMEGA-PRESS pulse sequence designed for this study. The slice selective and spectral selective 180$^{\circ }$ RF-pulses together with slice selective gradient pulses and spoiler gradients (indicated respectively as open trapezoidal and rectangular shapes) are applied according to the scheme in [13], where $t_2 = t_3 = t_4 = t_1 + t_5$. The two bipolar pairs of diffusion-weighting gradient pulses (shaded rectangular shapes) are superimposed to the spoiler gradients surrounding the slice selective 180$^{\circ }$ RF-pulses, and have a fixed duration of $\delta$, a varying intensity given by *G*, and a separation given by $\Delta$. Within each pair, the bipolar gradient pulses are separated by the time $\tau$. $\Delta _s$ indicate the evolution time for the spatial-dependent cross term between the applied gradients and spoiler gradients. As indicated, the diffusion-weighted gradients can in principle be applied simultaneously in all orthogonal directions, however they were only applied along the z-axis in this study

The dMEGA-PRESS sequence is presented in Fig. 1. Diffusion-weighting is introduced by superimposing bipolar gradients on the already existing spoiler gradients surrounding the slice selective 180$^{\circ }$ radiofrequency-pulses (RF-pulses) in the MEGA-PRESS encoding. All other parameters, including the timing of all RF-pulses, slice selective gradient pulses, and spoiler gradient pulses were applied according to the scheme presented in [13]. By superimposing the bipolar gradients on the spoiler gradients the other parameters, most importantly $t_1$–$t_5$ in Fig. 1, can be kept the same as is a regular MEGA-PRESS sequence. Bipolar gradients also reduce the influence of cross terms between gradient pulses in the diffusion attenuation [4, 6, 7]. At the minimum intensity of the diffusion-weighted gradients, i.e. only spoiler gradient intensity, the sequence corresponds to a regular MEGA-PRESS. The indicated positive polarity of the spoiler gradients could potentially limit the maximum achievable intensity of the diffusion-weighted gradients. However, since the required strength of the spoiler gradients is very low, this influence can safely be ignored.

The b value was calculated analytically according to the approach given in [14] and outlined in [6], which results in

1 $$\begin{aligned} b = \gamma ^2 (2 \delta ) ^2 G ^2 \cdot (\Delta + \frac{1}{2} \tau +\frac{4}{3} \delta ), \end{aligned}$$

where G denotes the diffusion gradient amplitudes causing diffusion-weighting attenuation. The detailed calculation of this b value is shown in supplementary materials Fig. S1, which also includes direction-dependent cross terms between the diffusion-weighting gradients and the spoiler gradients surrounding the editing RF-pulses. The diffusion time, $t_D =\Delta + \frac{1}{2} \tau +\frac{4}{3} \delta$. The attenuation caused solely by the spoiler gradients is constant throughout the experiment, and can therefore be treated as a constant offset, which is not included in the equation.

## Materials and methods

### Implementing the dMEGA-PRESS pulse sequence

The dMEGA-PRESS pulse sequence was implemented on two different instruments. Initial in vitro experiments were performed on phantom solutions using a 11.7 T Bruker Ascend 500 MHz vertical wide bore spectrometer. These experiments were used to validate the performance of the pulse sequence, both when it comes to spectral selective refocusing and diffusion-weighting. The method was then implemented on a 7.0 T Bruker Pharmascan 70/16 horizontal small animal MRI scanner. Due to an abrupt out-phasing of the animal scanner, in vivo experiments were prioritised, with very limited time for systematic experiments on phantom solutions, which is therefore not included. In both in vitro and in vivo experiments the total echo time was kept constant at 68 ms, which is optimal for MEGA-PRESS editing of GABA. To achieve full refocusing the time delays in Fig. 1 were: t2 = t3 = t4 = t1 + t5 = TE/4 = 17 ms [13, 15]. The bandwidth of the 180$^{\circ }$ slice selective pulses were 3.5 kHz (7.0 ppm) and 2.5 kHz (8.3 ppm) respectively for 11.7 T and 7.0 T. Shinnar–LeRoux pulses with a duration of 10 ms and bandwidth of 170 Hz, corresponding respectively to 0.34 ppm and 0.56 ppm at 11.7 T and 7.0 T, were used for spectral selective refocusing. Spoiler gradients were rectangular pulses with a duration of 3 ms and with an amplitude of 62 mT/m. Other time intervals were: $\delta = 3$ ms, $\tau = 1.5$ ms, $\Delta = 27$ ms, and $\Delta _s = 31.5$ ms. Due to the relatively large voxel size in the in vivo experiments the effects from anisotropy is expected to average out, and this, combined with the restriction of a 1 h time limit for animal scanning, diffusion gradients were applied only in the z-direction. Outer volume suppression was enabled using the built-in subroutines of the Paravision software. Local iterative shimming on the voxel was performed to the second-order, both in the in vitro and in vivo experiments. The shimming quality was determined by evaluating the peak of water or NAA at 2.0 ppm. Shimming gradients were adjusted until a full width at half maximum of less than 3 Hz for in vitro experiments and less than 12 Hz for in vivo experiments was achieved, ensuring optimal magnetic field homogeneity. A built-in algorithm was used for online evaluation of eddy currents, where a water reference scan was recorded before each single experiment for the purpose of eddy current correction.

### In vitro phantom study

In vitro experiments were performed on aqueous solutions of selected metabolites. All solutions were prepared using distilled water where the pH was adjusted to 7.4 using a phosphate buffer. Each solution was added to a 10 mm NMR tube. To examine the spectral editing performance of the dMEGA-PRESS pulse sequence, experiments were performed on a solution containing only GABA (5.0 mM) and NAA (20.0 mM). The pulse sequence was then applied to a series of solutions with varying concentration of GABA (2.5, 4.2, 5.9, 7.9 and 10.3 mM), and where the concentrations of the other metabolites were kept constant (in mM): Glu: 12.5, Cho 3.0, Cr 10.0, NAA 12.5, Lac 5.0, and Ins 7.5. This series of solutions are referred to as ‘metabolite phantoms’. The reliability of the diffusion-weighting was tested on the metabolite phantom containing 5.9 mM GABA.

All in vitro experiments were conducted at 25 $^{\circ }$C on a 11.7 T Bruker Ascend 500 MHz vertical wide bore spectrometer equipped with a commercial Bruker MicWB40 micro imaging probe, using ParaVision 6.0.1 software (Bruker BioSpin, Billerica, MA, USA). The maximum available gradient strength was 1.4 T/m. For placement of the voxel, the Paravision multi-slice localiser was utilised. The shimming was done automatically based on an acquired $B_0$-map. Water suppression was manually adjusted for each sample using VAPOR. A $4\times 4\times 4~\text {mm}^3$ voxel was placed in the middle of the 10 mm NMR tube. All spectra were acquired using a regular PRESS sequence or the dMEGA-PRESS sequence shown in Fig. 1, with respectively TE/TR = 16/3000 ms and TE/TR = 68/3000 ms. 4096 data points were acquired with 16 averages. Diffusion weighting was performed with a total of 9 b values in the range 0.01–4.19 $\text {ms}/\upmu \text {m}^2$. Two datasets were acquired for each b value, one ‘ON’ spectrum, where the spectral selective 180$^{\circ }$ RF-pulses were placed at 1.9 ppm, causing refocusing of the signals from GABA at 2.3 and 3.0 ppm (GABA-editing) [13], and the signals from Glu at 2.4 and 3.75 ppm (co-editing) [16]. In the ‘OFF’ spectrum, these pulses were placed at 7.5 ppm to acquire a spectrum without selective refocusing.

### In vivo animal study

In vivo experiments were performed on adult Wistar rats (N = 10, 5 male and 5 female). Animal experiments were conducted according to ethical guidelines at University of Bergen. The animals were anaesthetised with isoflurane (2.5 vol% added to 1:2 oxygen:nitrogen). Respiration was monitored and a physiological temperature (37.0 ± 0.2 $^{\circ }$C) was maintained by a heater module (SA instruments Inc, NY, USA). After positioning the animal in the scanner, isoflurane was reduced to maintain a respiration level of 60–70 breaths min$^{-1}$ (1.6–2.0 vol%). Two animals were excluded (one male, one female) due to problems with water suppression during scans. All animal experiments were performed on a 7.0 T Bruker Pharmascan 70/16 horizontal small animal MRI scanner with software ParaVision 6.0.1 (BrukerBioSpin, Billerica, MA, USA) and a 55 mm/23 mm tx/rx quad head coil (Bruker Corporation, Model No: MT0206). The maximum available gradient strength was 0.7 T/m. The animal was fixed using a standard Bruker animal cradle.

**Fig. 2:**
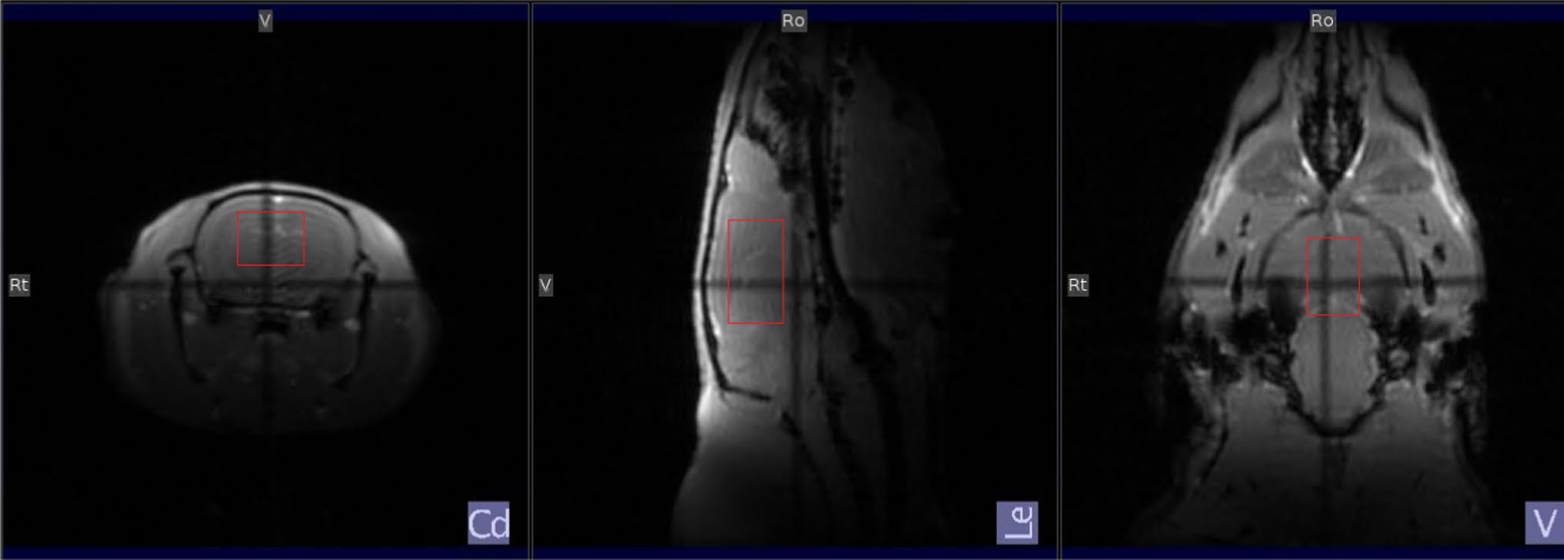
Placement of a $7\times 4\times 7~\text {mm}^3$ voxel in the central region of a rat brain, based on the multi-slice localiser image

Localisation was performed based on a multi-slice localiser image with FOV = $50\times 50$ mm$^2$. An $8\times 5\times 8$ mm$^3$ voxel was chosen for first, second, and third order shimming. The shimming was done automatically based on an acquired $B_0$-map. For localised $^1$H spectroscopy a $7\times 4\times 7$ mm$^3$ voxel, shown in Fig. 2, was placed in the middle of a heterogenous region of the brain, including mid brain, cortex, thalamus and hippocampus, averaging out regional concentrations and diffusion properties of metabolites [17]. As described above, local iterative shimming on the voxel was performed until a full width at half maximum for water and/or NAA of less than 12 Hz was achieved, which is within the recommended limit for animal MRS in the rodent brain [18]. The voxel was placed at a safe distance from the edges of the skull to avoid any susceptibility artefacts.

MR spectra were acquired using the dMEGA-PRESS sequence with TE/TR = 68 ms/3000 ms. 4096 data points were acquired with 32 averages. Water suppression was achieved using VAPOR, with manually optimised RF attenuations. ‘ON’ and ‘OFF’ diffusion-weighted spectra were acquired using the same settings for the spectral selective 180$^{\circ }$ RF-pulses as described for the in vitro experiments. Diffusion weighting was performed with a total of 5 b values in the range 0.03–4.38 $\text {ms}/\upmu \text {m}^2$, and the gradient pulses were applied along the z-direction in all experiments. A navigator was used for online frequency drift, which can be more pronounced when performing diffusion experiments.

### Processing

Eddy current correction of in vitro and in vivo data were performed using ParaVision 6.0.1 software (Bruker BioSpin, Billerica, MA, USA), where the procedure is based on an accumulated signal from a water reference scan, resulting in a signal which is demodulated based on the temporal phase evolution of the strongest spectral component of this reference scan. Averaging, combination of spectra, and frequency drift correction, was performed online in ParaVision. Further processing was performed using an in-house script made in MatLab 2019b (MathWorks inc., Massachusetts), where the raw data were first combined and averaged after correcting for frequency drifts that might have occurred during the experiment. The data were then zero filled to $2^{15}$ to achieve a Hz/pt resolution that follows the standard in the GANNET pipeline [19] used in the subsequent processing. Global phase correction was performed using the ‘Automated phase Correction based on Minimization of Entropy’ (ACME) algorithm [20]. Phase correction was performed in two steps, first using the water peak, then using the chosen region of interest corresponding to the metabolite being investigated. This approach resulted in a more robust phase correction. Edited spectra, which enables isolation and quantification of molecules with refocused signals, were obtained for each b value by subtracting the ‘OFF’ from the ‘ON’ spectrum.

Spectral fitting and deconvolution [6] is often performed using a basis set representing linear combinations of individual metabolite spectra at a given experimental condition [21, 22]. A basis spectrum for a specific metabolite can be generated from separate experimental data or through simulations based on density matrix theory and input of experimental conditions [6]. When analysing edited spectra a simpler approach with lineshape peak fitting and integration of each individual signal is common, where the use of GANNET [19] on data from GABA-editing is an example. An analysis based on lineshape peak fitting and integration was applied in our study, where each individual peak of interest were curve fitted using in-house algorithms inspired by the GANNET pipeline [19] and LWFIT [23]. Signal intensities of the different metabolites at varying diffusion-weightings were then obtained from integration of the curve fitted peaks. This corresponds to a “no prior knowledge” approach to spectral analysis, allowing for larger flexibility compared to methods where the spectral data are fitted using basis sets obtained from simulations or separate experiments [23]. This approach allows for the use of the same analytical method for the ‘OFF’, ‘ON’ and edited diffusion-weighted data, enabling a direct and simple comparison of the improved quality of the obtained ADC values for specific metabolites. Fit errors for the spectral analysis of each metabolite was calculated according to the procedure used in GANNET pipeline [19].

Details regarding hardware, acquisition, data analysis, and data quality are given in Supporting Information, Table S1.

## Results

### In vitro

**Fig. 3:**
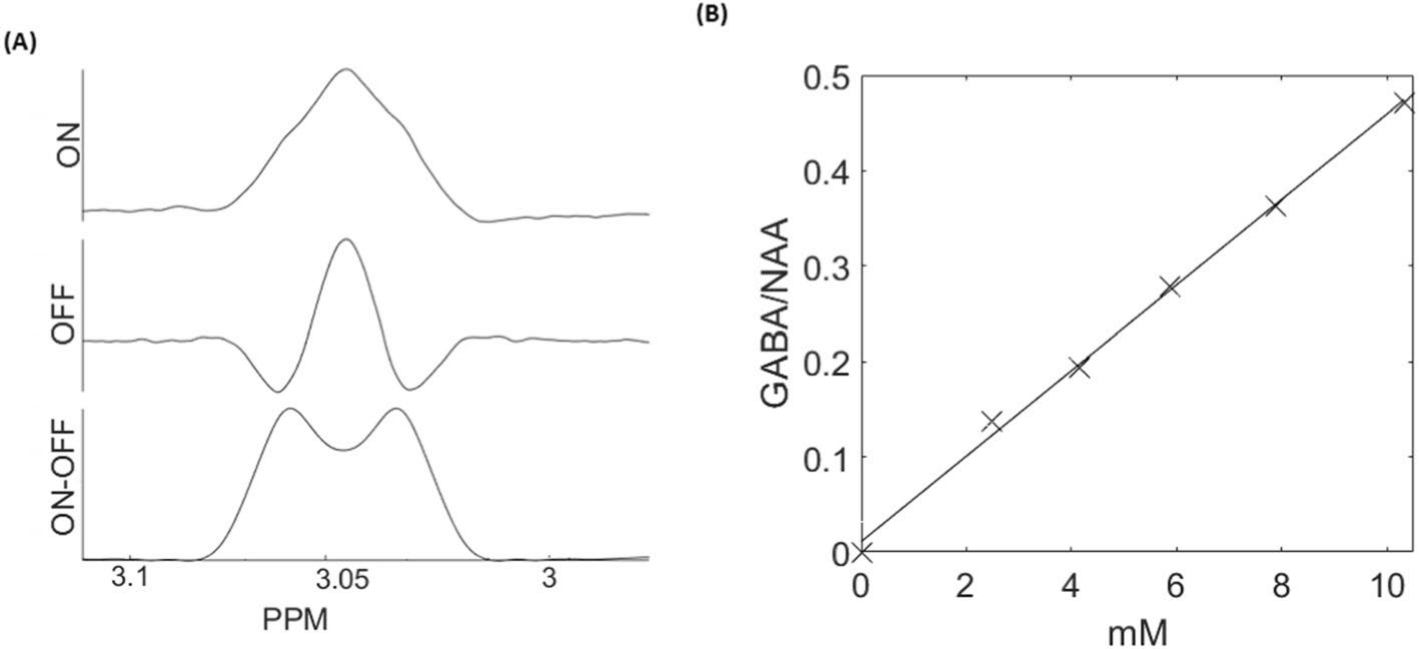
**A** Gaba editing in the GABA + NAA solution. Top: ‘OFF’-spectrum, middle: ‘ON’-spectrum, bottom: edited spectrum. **B** Calibration curve in a series of metabolite phantoms having varying GABA concentrations, with respect to the edited GABA signal at 3.0 pppm

As shown in Fig. 3A, applying the dMEGA-PRESS sequence on the solution with GABA and NAA, results in the expected shapes of the ‘OFF’, ‘ON’ and edited (‘ON’–‘OFF’) spectra for the GABA peak at 3.0 ppm. The calibration curve obtained from a series of metabolite phantoms, presented in Fig. 3B, shows a linear response between concentration and relative intensity of the edited GABA signal, verifying the dMEGA-PRESS quantification capabilities. These data are obtained using an insignificant strength of the diffusion-weighting gradient pulses, i. e. a regular MEGA-PRESS.

**Fig. 4:**
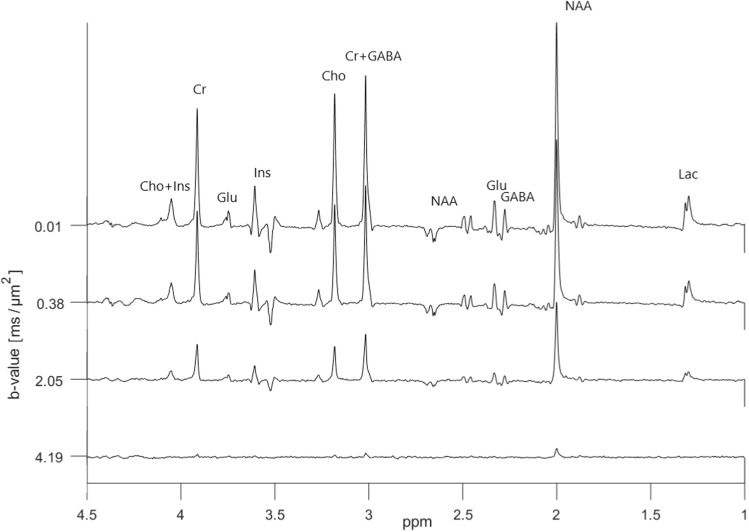
In vitro diffusion-weighted ‘OFF’ spectra acquired using the dMEGA-PRESS sequence in the metabolite phantom with a GABA concentration of 5.9 mM. A total of 9 b values were varied in the range 0.01–4.19 $\text {ms}/\upmu \text {m}^2$. A selection of four spectra is shown here for clarity. The spectral selective 180$^{\circ }$ pulses are set to 7.5 ppm

Diffusion-weighted ‘OFF’ spectra from the metabolite phantom having a GABA concentration of 5.9 mM are presented in Fig. 4. These data corresponds to spectra obtained using a regular DW-PRESS sequence with a relatively long echo time and potentially large J-modulations.

**Fig. 5:**
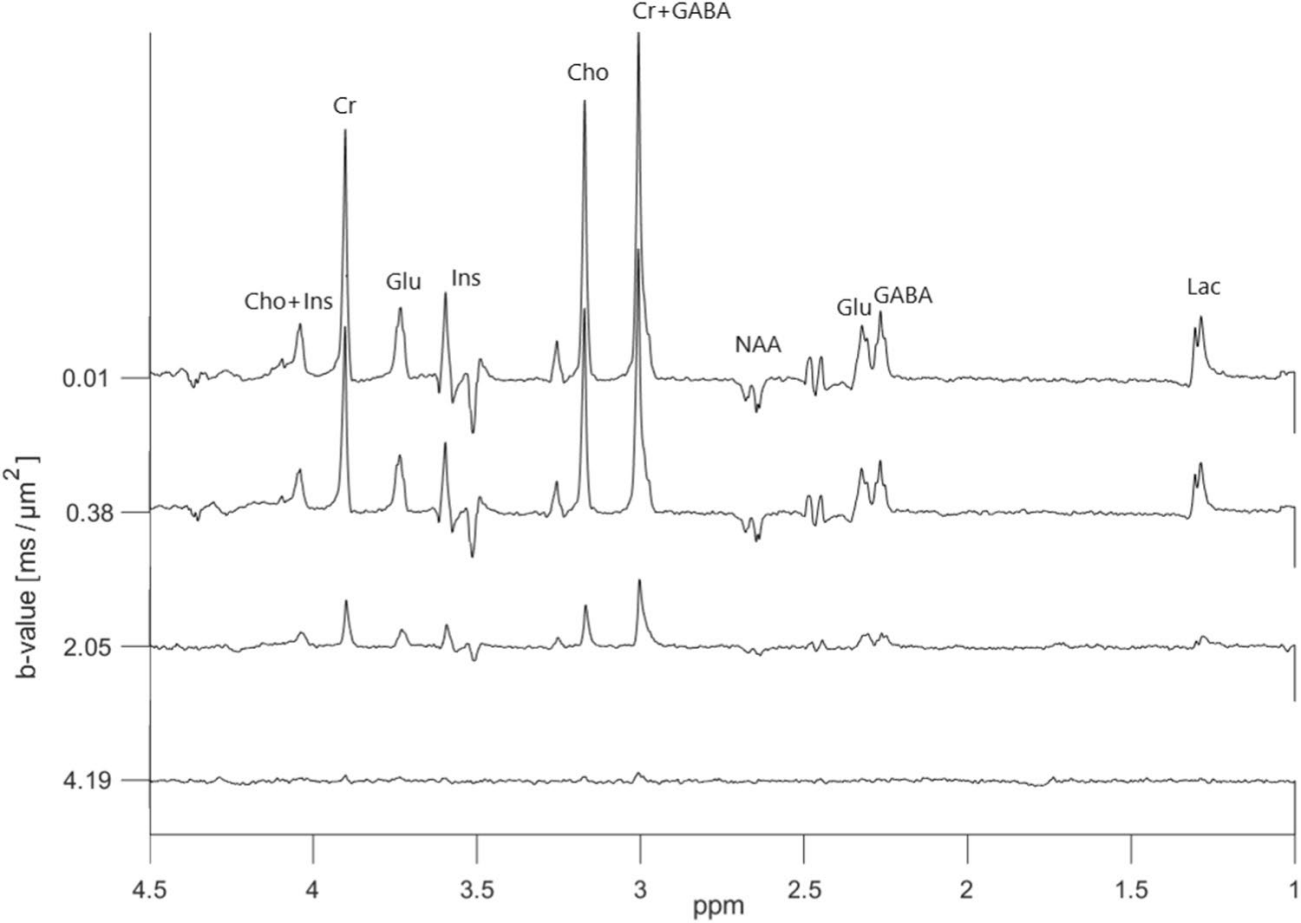
In vitro diffusion-weighted ‘ON’ spectra acquired using the dMEGA-PRESS sequence in the metabolite phantom with a GABA concentration of 5.9 mM. A total of 9 b values were varied in the range 0.01–4.19 $\text {ms}/\upmu \text {m}^2$. A selection of four spectra is shown here for clarity. The spectral selective 180$^{\circ }$ pulses are set to 1.9 ppm

**Fig. 6:**
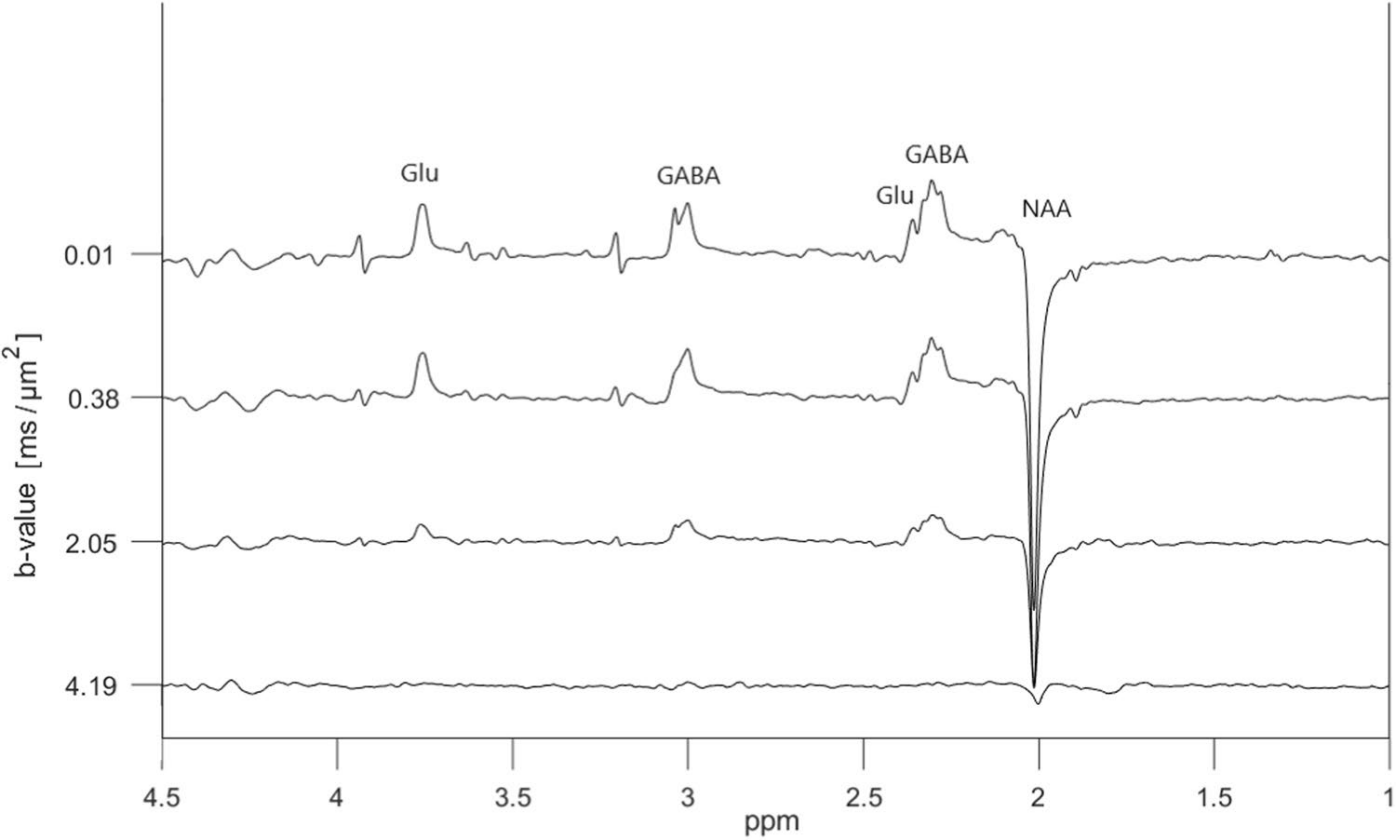
In vitro diffusion-weighted edited spectra (‘ON’–‘OFF’) obtained from the spectra shown in Figs. 4 and 5

The corresponding ‘ON’ spectra from the same metabolite phantom, presented in Fig. 5, result in refocused peaks of GABA at 2.3 ppm and 3.0 ppm, and Glu at 2.4 and 3.75 ppm. The edited (‘ON’–‘OFF’) diffusion weighted spectra are shown in Fig. 6. The only signals of significance are those from GABA, Glu and NAA, as expected from a GABA-edited MEGA-PRESS experiment. In addition to the refocused and edited peak of GABA at 3.0 ppm, we have in the following analysis chosen to focus on the refocused and edited peak of Glu at 3.75 ppm since this is the peak with least spectral overlap.

**Fig. 7:**
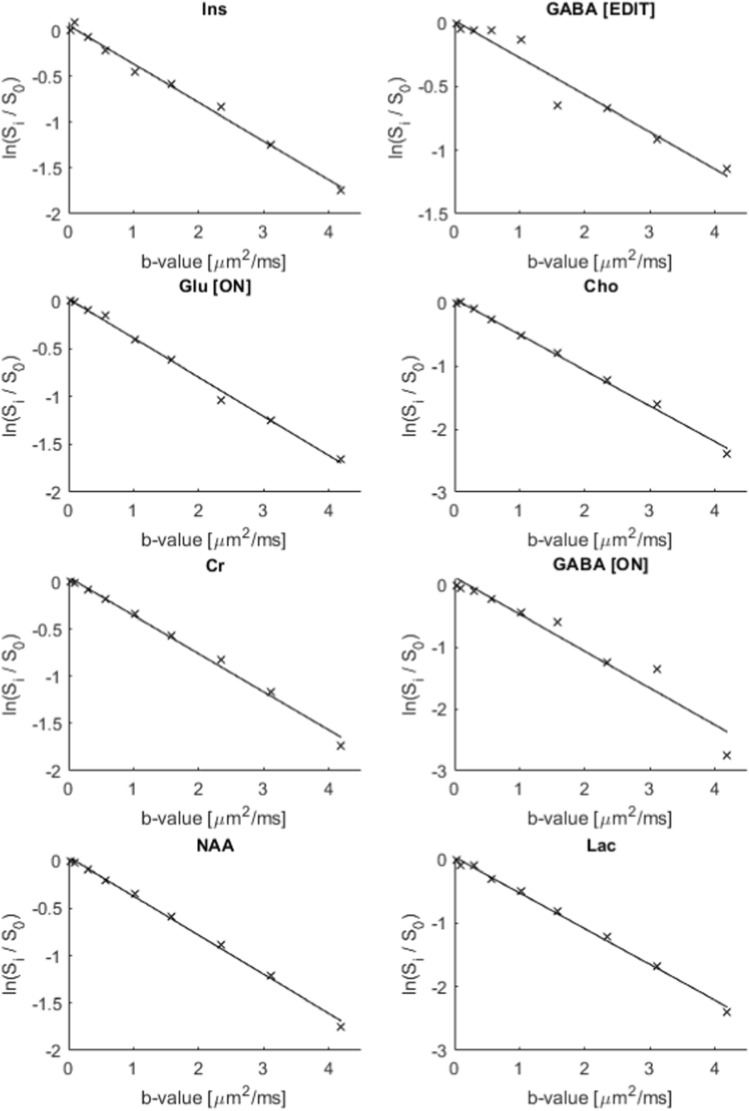
Plots of $ln(S_i/S_0)$ versus b values for selected peaks in spectra obtained from the metabolite phantom, with linear regression to determine the ADC-value. All plots are derived from the ‘OFF’ spectra apart from GABA [ON], Glu [ON], and GABA [EDIT]

The diffusivity of water in an unsuppressed-water-experiment was calculated to be $2.30 \pm 0.02~\upmu \text {m}^2/\text {ms}$ at 25 $^{\circ }$C. The analysis based on lineshape peak fitting of selected signals resulted in a fit error (obtained from the GANNET pipeline) of less than 20% for all the peaks and in all spectra obtained with increasing b values. Selected logarithmic plots of diffusion-weighted signal intensities versus b values are presented in Fig. 7. A linear relationship is achieved for all the metabolite signals of interest. Notably, the attenuations for the GABA[edit] signal at 3.0 ppm, and also to some degree for the GABA[ON] signal at 2.3 ppm, are more noisy. ADC-values obtained from the linear regressions are presented in Table 1. Within the limits of uncertainties the ADC values for Cr, Cho, Ins, and Lac obtained from ‘OFF’ and ‘ON’ spectra are in good agreement, as expected from metabolites with signals not influenced by the editing pulses. ADC-values obtained from the GABA peak at 2.3 ppm in the ‘ON’ and edited spectra are in good agreement, however with higher uncertainties in the edited spectra. In comparison a slightly higher ADC-value is obtained from the edited GABA peak at 3.0 ppm. In general the uncertainties are higher in all the values obtained from the edited diffusion weighted spectra. Notably, the highest uncertainty can be found in the ADC value for the Glu peak at 3.75 ppm in the ‘OFF’ spectra due to the strong influence from J-modulation, while reliable values were obtained from this peak in the ‘ON’ and edited spectra where this peak is fully refocused.

**Table 1:** Obtained ADC values of metabolites in the metabolite phantom at $25\,^{\circ }$C (value ± SE) $[\upmu \text {m}^2/\text {ms}]$

	OFF	ON	EDIT
Lac [1.4]	0.86 ± 0.05	0.86 ± 0.05	–
NAA [2.0]	0.68 ± 0.01	–	0.66 ± 0.02
GABA [2.3]	–	0.83 ± 0.04	0.84 ± 0.07
GABA [3.0]	–	–	0.88 ± 0.04
Cr [3.0]	0.83 ± 0.02	0.82 ± 0.02	–
Cho [3.2]	0.99 ± 0.03	0.97 ± 0.03	–
Ins [3.6]	0.80 ± 0.02	0.80 ± 0.02	–
Glu [3.75]	0.7 ± 0.1	0.70 ± 0.01	0.68 ± 0.04

The values and corresponding standard errors (SE) are obtained from the linear regressions visualised in Fig. 7. The numbers in brackets indicate the chemical shift of the signal used

### In vivo

From the edited dMEGA-PRESS spectra with insignificant gradient strengths, using the GANNET pipeline [19], and referenced to an unsuppressed-water-experiment on the same subject, the mean concentration of GABA in the 8 test subjects was determined to be $(2.5 \pm 0.4)$ mM.

**Fig. 8:**
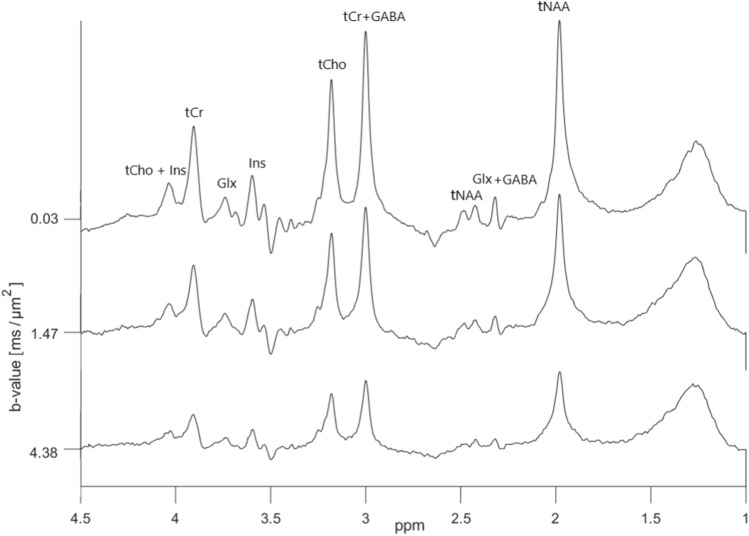
In vivo diffusion-weighted ‘OFF’ spectra obtained using the dMEGA-PRESS sequence in a rat brain with b values in the range 0.03-4.38 $\text {ms}/\upmu \text {m}^2$. For clarity, only 3 out of 5 spectra are shown. The spectra are obtained in a $7\times 4\times 7~\text {mm}^3$ voxel, as indicated in Fig. 2

**Fig. 9:**
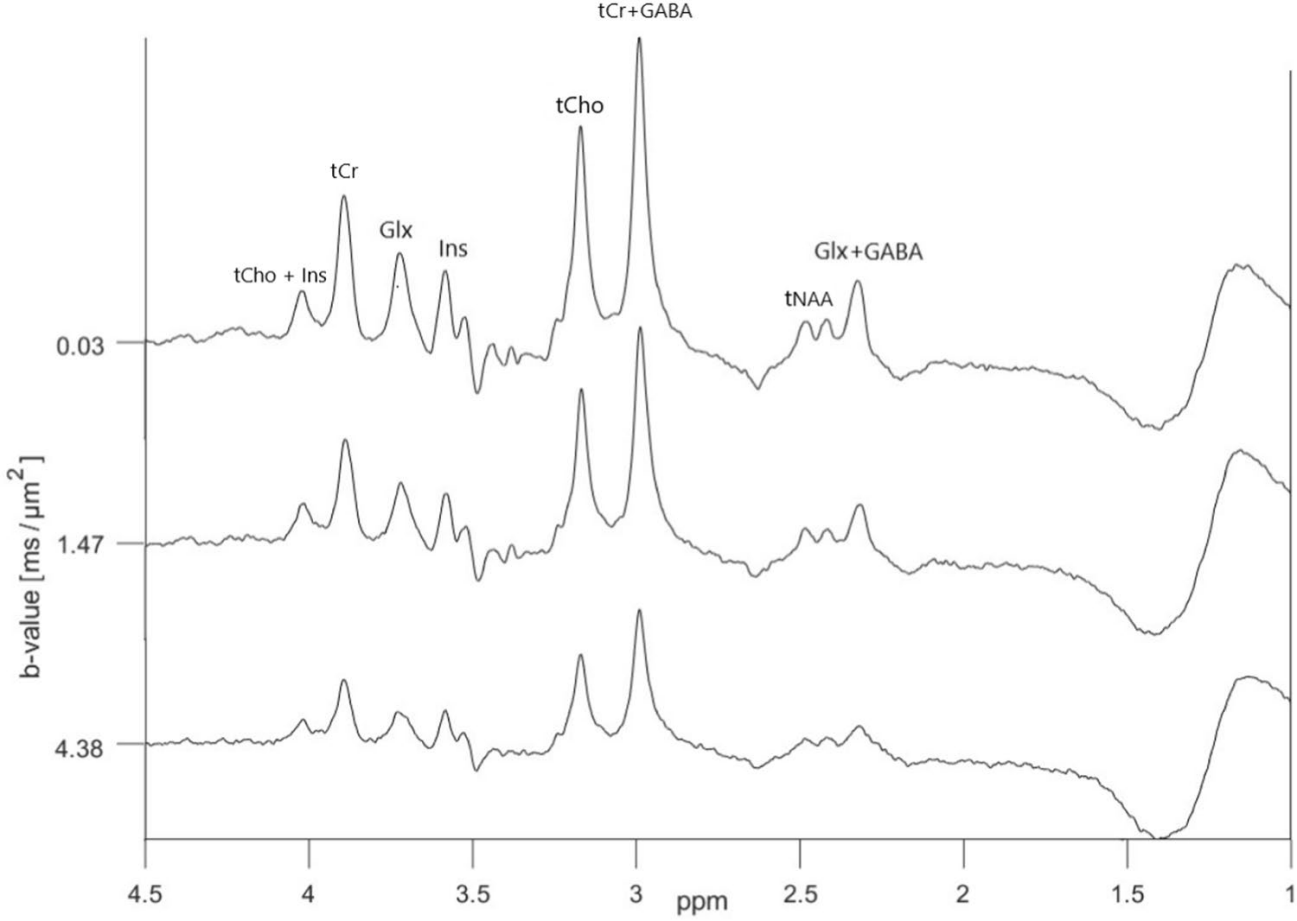
In vivo diffusion-weighted ‘ON’ spectra obtained using the dMEGA-PRESS sequence in a rat brain with b values in the range 0.03–4.38 $\text {ms}/\upmu \text {m}^2$. For clarity, only 3 out of 5 spectra are shown. The spectra are obtained in a $7\times 4\times 7~\text {mm}^3$ voxel, as indicated in Fig. 2

**Fig. 10:**
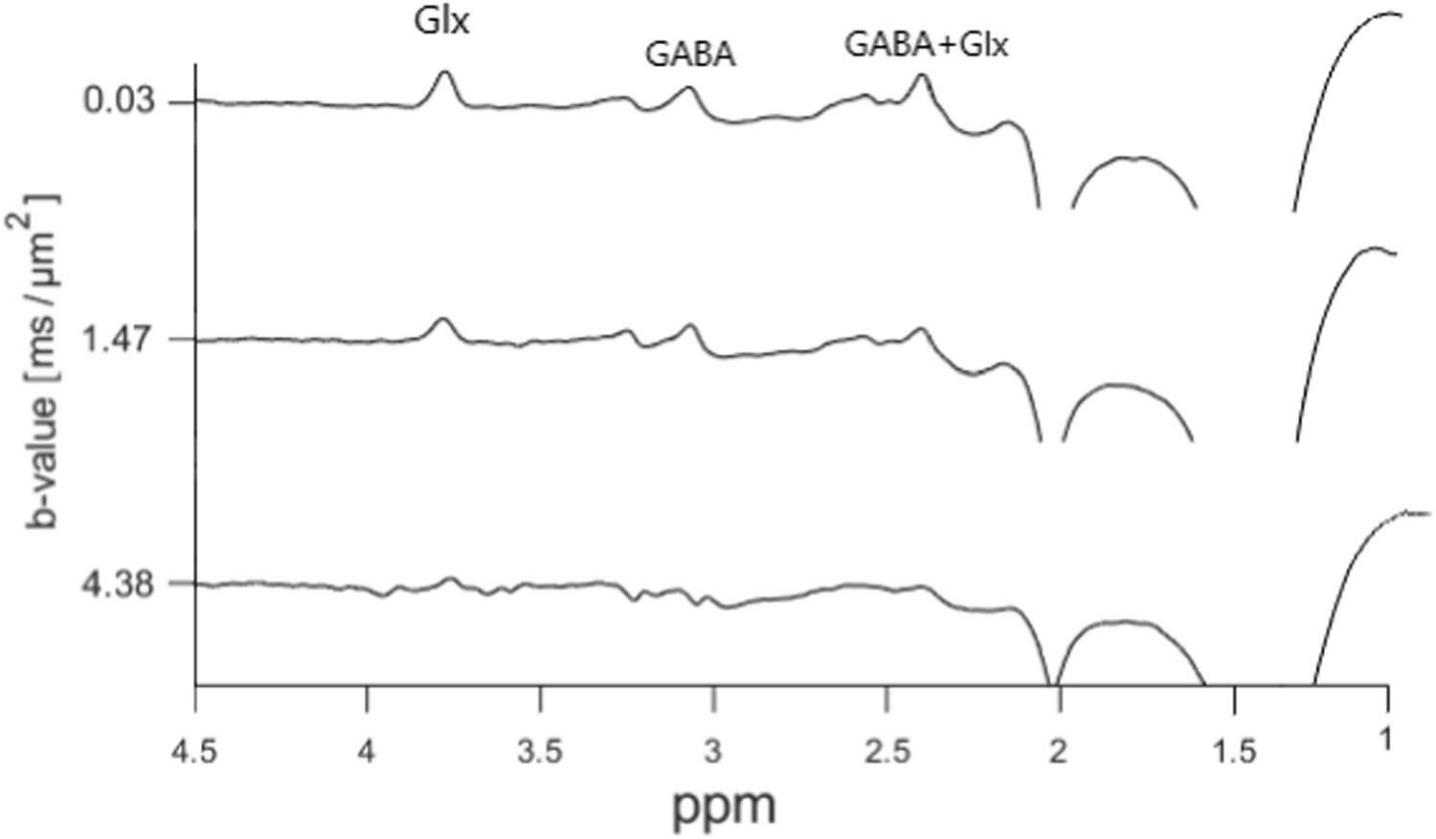
In vivo diffusion-weighted edited spectra obtained subtracting the ‘ON’ spectra in Fig. 9 from the ‘OFF’ spectra in Fig. 8

Diffusion-weighted ‘OFF’ spectra obtained in the central cortex in one of the subjects are shown in Fig. 8. The broad peak located around 1.3 ppm is due to lipid signals, which unfortunately were present in all spectra from all subjects, even though OVS with sufficient saturation bands was applied. Since our analysis is based on a simple lineshape peak fitting for individual peaks, the signals from glutamate and glutamine could not be deconvoluted, and are labelled as Glx. Notice the significant J-modulations and low intensity in overlapping peaks from GABA/Glx located at 2.3–2.4 ppm, and the peak from Glx located at 3.75 ppm. In the corresponding ‘ON’ spectra shown in Fig. 9 these signals are refocused and have more pronounced peaks. The edited (‘ON’–‘OFF’) diffusion-weighted spectra are shown in Fig. 10. The significant peaks are from GABA and Glx, but minor signals from tCr (3.9 ppm) and tCho (3.2 ppm) are also present. Also in the ‘ON’ spectra lipid signals were present around 1.3 ppm, and here with some significant phase distortions, resulting in non-eliminated and significant contributions from these signals in the edited spectra in this area.

The analysis based on lineshape peak fitting of selected signals resulted in a fit error (obtained from the GANNET pipeline) of less than 20% for all the peaks and in all spectra obtained with increasing b values. Examples of results from the peak fitting are presented in Figures S2 and S3 in Supporting Information.

**Fig. 11:**
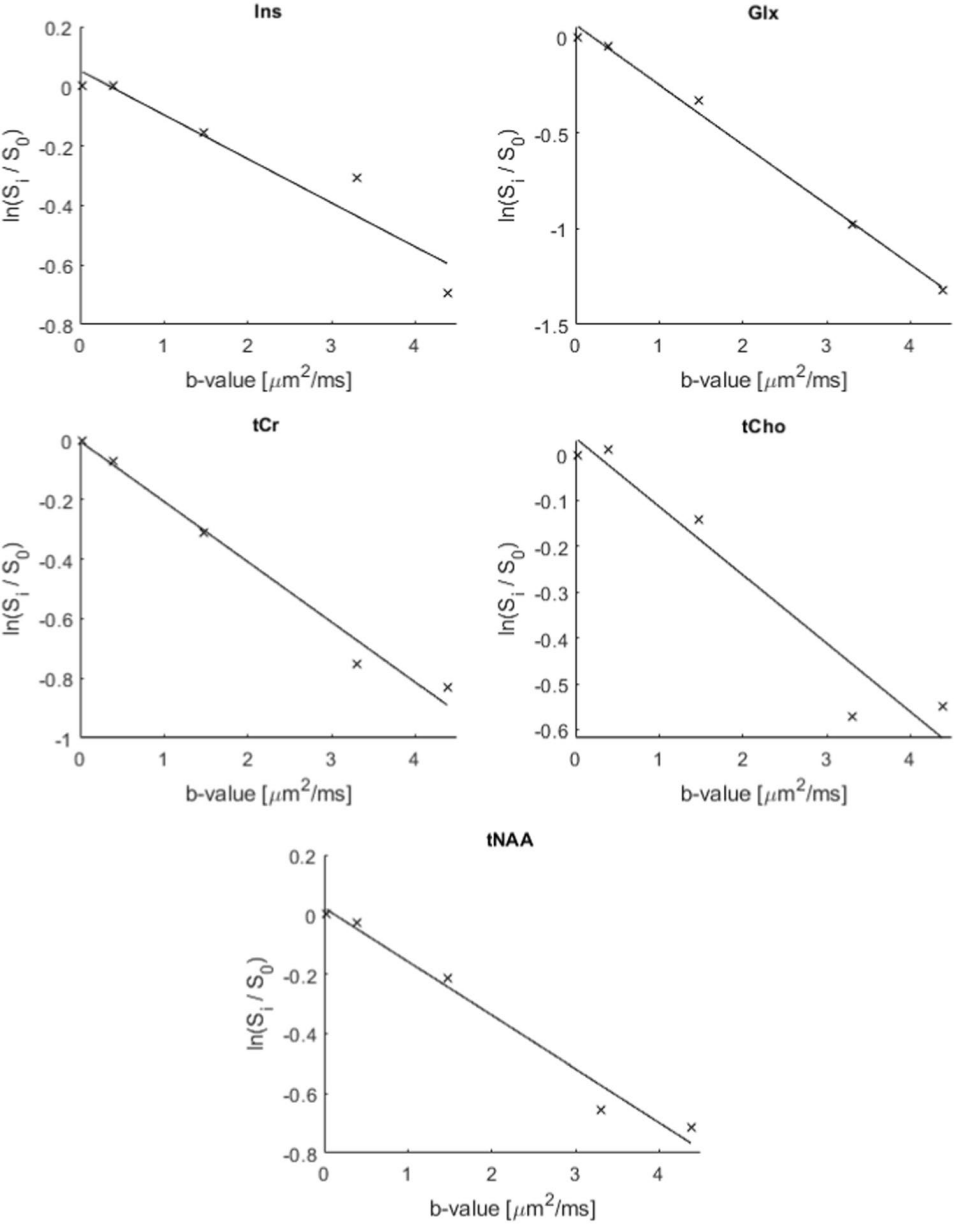
Plots of $ln(S_i/S_0)$ versus b values for selected peaks in the spectra presented in Figs. 8, 9 and 10. The plots are from one subject, and does not represent the full dataset. All plots are derived from the ‘OFF’ spectra apart from Glx which is from the ‘ON’ spectra

Logarithmic plots of diffusion-weighted signal intensities versus b values for selected peaks in one of the test subjects are presented in Fig. 11. Estimated ADC-values obtained from ‘OFF’, ‘ON’ and edited diffusion-weighted spectra are presented in Table 2. Based on the overlapping standard deviations there are no significant differences between the ADC-values of individual metabolites obtained from the different sets of spectra, even though the values for both tCho and Ins are higher in the ‘ON’ spectra compared to ‘OFF’ spectra. It was not possible to obtain reliable ADC values for GABA from edited spectra, nor from Glx in the ‘OFF’ spectra. However, as seen in the plots in Fig. 11 and the ADC values presented in Table 2, the data for Glx at 3.75 ppm in the refocused ‘ON’ and the edited spectra were of the same quality as for the other metabolites.

**Table 2:** Estimated ADC values of metabolites in the rat brain (mean ± SD) $[\upmu \text {m}^2/\text {ms}]$

	OFF	ON	EDIT
tNAA [2.0]	0.13 ± 0.01	–	–
tCr [3.0]	0.17 ± 0.04	0.18 ± 0.03	–
tCho [3.2]	0.14 ± 0.02	0.18 ± 0.04	–
Ins [3.6]	0.12 ± 0.06	0.18 ± 0.07	–
Glx [3.75]	–	0.16 ± 0.02	0.14 ± 0.05

The mean values and standard deviations (SD) are obtained from the variation in the values obtained from the different subjects. The numbers in brackets indicate the chemical shift of the signal used

## Discussion

The spectral refocusing and corresponding editing experiments performed on the metabolite phantom (Fig. 3B) verified a linear correlation between the signal intensity of the edited peak at 3.0 ppm and the concentration of GABA. These data were obtained at minimum values of the diffusion-weighting gradients, and therefore corresponds to a regular MEGA-PRESS experiment. Analysis of the corresponding in vivo MEGA-PRESS data was resulted in an average GABA concentration of $(2.5 \pm 0.4)$ mM, which is in agreement with previous MRS studies in rodents [24–28]. To our knowledge, the MEGA-PRESS sequence has only been implemented on preclinical systems in two previous studies, one study in rats [25] at 4.7 T, and one study in mice [15] at 9.4 T. The quality of our edited data, as indicated in the standard deviation of the obtained GABA concentrations, is at the same level as observed in these two previous studies.

The signals from GABA and Glu are strongly J-modulated in the diffusion-weighted in vitro ‘OFF’ spectra, making it impossible to obtain a reliable ADC-value for GABA, and resulting in a high uncertainty in the ADC value for Glu. The uncertainty in the ADC value for Glu based on the peak at 3.75 ppm is reduced in the ‘ON’ and ‘edit’ data compared to the analysis based on the ‘OFF’ data. Furthermore, a reliable ADC value of the GABA signal at 3.0 ppm was obtained from the diffusion-weighted edited spectra. In general, the ADC values from the edited spectra have higher uncertainties, most likely an effect caused by spectral subtraction. The obtained ADC values (Table 1) are consistent with data from other in vitro studies [9, 10, 29] when correcting for differences in sample temperature using the equations for interpolation given in [30]. A literature value for GABA in phantom solutions was not available, but from separate diffusion measurements performed on a liquid state NMR spectrometer in our lab, a diffusion coefficient of $0.85 \pm 0.02~\upmu \text {m}^2/\text {ms}$ was obtained for GABA at 25 $^{\circ }$C. This value, together with values obtained in corresponding separate diffusion measurements of all the other metabolites are all in good agreement with the ADC values obtained in Table 1. The obtained ADC value of water ($2.30 \pm 0.02~\upmu \text {m}^2/\text {ms}$ at 25 $^{\circ }$C), is in very good agreement with the established literature value [30].

The in vivo data show that selective refocusing leads to an increase in the intensities of the signals of GABA and Glx at respectively 2.3 ppm and 2.4 ppm, and the signal from Glx at 3.75 ppm, as expected in a MEGA-PRESS optimised for GABA editing. An ADC value for Glx with a relatively low uncertainty was therefore determined from the ‘ON’ and edited spectra, but was not obtainable from the ‘OFF’ spectra. One of the goals of this study was to enable a reliable estimation of the in vivo ADC value of GABA through the use of full MEGA-PRESS editing. However, the analysis of the edited GABA peak at 3.0 ppm (Fig. 10) resulted in unstable and noisy decay curves in all the subjects, and ADC values based on this edited signal was not obtainable. This could be caused by errors introduced when performing spectral subtraction of in vivo spectra, which could also explain the higher uncertainty in the ADC values for Glx obtained from the edited spectra. Furthermore, using MEGA-PRESS, signals from macromolecules (MM) at 3.0 ppm are coedited through the influence of the ‘ON’ editing pulse on the MM peak at 1.7 ppm, which potentially has an impact on the diffusion analysis of the edited GABA peak. In future studies signal from MM could be suppressed by moving the editing pulse in the ‘OFF’ spectra from 7.5 to 1.5 ppm, resulting in equal degree of refocusing on the MM signal at 3.0 ppm in the ‘ON’ and ‘OFF’ spectra, while GABA is still only refocused in the ‘ON’ spectra. The MM signal is then eliminated in the edited spectra, while the signal from GABA remains [31]. Another possible approach for elimination of the MM signal is to use pre-inversion pulse to obtain a separate MM spectrum, which can be subtracted from the total metabolite spectrum [32], however this method is time consuming and more prone to subtraction errors. Both approaches can be implemented in the dMEGA-PRESS sequence, but this was not attempted here.

In addition to the ADC value based on the edited peak at 3.0 ppm in the metabolite phantom, the spectral resolution combined with signal refocusing also allowed for an estimation of an ADC value for GABA based on the peak at 2.3 ppm in the ‘ON’ and edited spectra. Due to the strong spectral overlap between GABA and Glx signals in this region of the in vivo spectra, an analysis based on lineshape peak fitting and integration was not possible. With a different analysis method it may be possible to also take advantage of the increased signal intensity of GABA and Glx in this spectral region, and this could be pursued further in future studies.

The limiting bandwidth of the volume selective 180$^ {\circ }$ refocusing rf-pulses and resulting B$_1$ inhomogeneities, leads to chemical shift displacement errors (CSDE) that causes selection of different voxel locations for different moieties of a molecule. In the consensus paper of Choi et al. [12], simulations performed using a field strength of 3.0 T and a refocusing bandwidth of 1.0 kHz showed that due to CSDE in the OFF spectrum, the editing efficiency for Lac and GABA will be reduced by respectively 50 and 20%. Comparing these simulations with our in vivo data obtained at 7.0 T and a refocusing bandwidth of 2.5 kHz, both being a factor of 2.5 higher than in [12], the loss of editing efficiency of GABA in our measurements can also be estimated to 20%. In [12] the effect on Glx was not evaluated, but simply assuming that the loss of editing efficiency scales linearly with the chemical shift difference between the editing rf-pulse and the target resonance, the loss in editing efficiency can be estimated to be around 30% for Glx in our measurements. This will have an impact of the estimated intensities of signals in the ‘OFF’ and edited spectra, and might influence the detectability of the ADC of GABA in vivo. However, this effect can be expected to be the same for all b values in the OFF and edited spectra, and should not have a significant impact on the estimated ADC values.

Despite the use of OVS in our acquisitions, all the in vivo spectra have contributions from lipid signals appearing as a relatively broad peak around 1.5 ppm with an inverted phase in the ‘ON’ spectra. This is a consequence of CSDE. The use of adiabatic pulses and other more specialised lipid suppression approaches [33] could reduce these artefacts. The presence of remaining lipid signals could potentially influence the diffusion analysis. However, peaks were individually curve fitted and integrated for each metabolite, making our analysis less susceptible to influences from lipid signals in other spectral areas, for instance baseline distortions that can influence an analysis based on basis sets. The disadvantage of analysing each peak individually is that overlapping signals cannot be resolved. However, the obtained in vivo ADC values have relatively low uncertainties and correspond well with values obtained in similar studies, indicating insignificant errors due to spectral overlap in our analysis.

The in vivo attenuations (Fig. 11) are mono-exponential for all of the metabolite signals. This is expected with relatively low gradient strengths, moderate b values with a maximum of approximately 4.5 $\text {ms}/\upmu \text {m}^2,$ and relatively short diffusion times, applied in this study, making the measurement less sensitive to compartmentalisation and non-Gaussian behaviour [6, 7, 34, 35]. Taking advantage of the maximum gradient strength on a preclinical scanner it is possible to reach higher b values using the dMEGA-PRESS sequence, but this was not pursued in this study, where the purpose was to demonstrate the feasibility of combining diffusion-weighting with selective refocusing and spectral editing.

Combined diffusion-weighting and selective refocusing can also be introduced to editing sequences with adiabatic pulses, like MEGA-LASER or MEGA-sLASER. In addition to GABA and Glx, examples of metabolites that would benefit from spectral refocusing are Ins, Glutathione, and 2-hydroxyglutarate.

In a recent paper [10] DW-SPECIAL and STE-LASER was compared in a dMRS study on rats using similar instrumentation as in our study. The obtained data showed that the shorter echo time in DW-SPECIAL (18 ms) compared to STE-LASER (33 ms) resulted in reduced uncertainty in the ADC values of strongly J-modulated metabolites. All the ADC values obtained in our study corresponds very well with the values obtained in [10]. In particular, the ADC value of Glx and its uncertainty obtained using DW-SPECIAL [10] were similar to our data.

The quality of the data allowed for a successful diffusion analysis on the edited GABA signal in the metabolite phantom, but not for the corresponding edited signal in the in vivo data. This is clearly one of the limitations when using dMEGA-PRESS, which rely on spectral subtraction of in vivo spectra. The total scanning time it takes to acquire ‘OFF’ and ‘ON’ spectra, and the fact that in the ‘ON’ spectra part of the spectrum is distorted by the spectral selective pulse, are other limitations worth emphasising.

## Conclusion

We have implemented and tested a dMEGA-PRESS sequence, where selective refocusing and spectral editing in MEGA-PRESS is combined with diffusion-weighting gradients to enhance and isolate signals from metabolite that are strongly influenced by J-modulations. The method was successfully applied to improve the quality of dMRS of GABA and Glu in a metabolite phantom and of Glx in rat brains. In general, the method is suitable for any metabolite that benefits from selective refocusing and spectral editing.

## Supplementary Information

Below is the link to the electronic supplementary material.

Supplementary file 1 (pdf 97 KB)

Supplementary file 2 (pdf 210 KB)

Supplementary file 3 (pdf 53 KB)

Supplementary file 4 (pdf 45 KB)

## Data Availability

Data presented as figures and tables in this article are available upon request.

## References

[1] Baliyan V, Das C, Sharma R, Gupta A (2016) Diffusion weighted imaging: technique and applications. World J Radiol 8(9), 785-798 10.4329/wjr.v8.i9.785 PMC5039674 27721941

[2] Basser P (1995) Inferring microstructural features and the physiological state of tissues from diffusion-weighted images. NMR Biomed 8(7–8):333–44 8739270 10.1002/nbm.1940080707

[3] Nicolay K, Braun K, Graaf R, Dijkhuizen R, Kruiskamp M (2001) Diffusion NMR spectroscopy. NMR Biomed 14(2):94–111 11320536 10.1002/nbm.686

[4] Ronen I, Valette J (2016) Diffusion-weighted magnetic resonance spectroscopy. eMagRes 131(2):733–750

[5] Cao P, Wu E (2017) In vivo diffusion MRS investigation of non-water molecules in biological tissues. NMR Biomed 30(3):3481 10.1002/nbm.3481 26797798

[6] Ligneul C, Najac C, Döring A, Beaulieu C, Branzoli F, Clarke W, Cudalbu C, Genovese G, Jbabdi S, Jelescu I, Karampinos D, Kreis R, Lundell H, Marjańska M, Möller H, Mosso J, Mougel E, Posse S, Ruschke S, Simsek K, Szczepankiewicz F, Tal A, Tax C, Oeltzschner G, Palombo M, Ronen I, Valette J (2023) Diffusion-weighted MR spectroscopy: consensus, recommendations, and resources from acquisition to modeling. Magn Reson Med 91(3):860-885 10.1002/mrm.29877 37946584

[7] Palombo M, Shemesh N, Ronen I, Valette J (2018) Insights into brain microstructure from in vivo DW-MRS. Neuroimage 182:97–116 29155183 10.1016/j.neuroimage.2017.11.028

[8] Nicolay K, Braun K, deGraaf R, Dijkhuizen R, Kruiskamp M (2001) Diffusion NMR spectroscopy. NMR Biomed 14(2):94–111 11320536 10.1002/nbm.686

[9] Landheer K, Schulte R, Geraghty B, Hanstock C, Chen A, Cunningham C, Graham S (2017) Diffusion-weighted J-resolved spectroscopy. Magn Reson Med 78(4):1235–1245 27797114 10.1002/mrm.26514

[10] Mosso J, Simicic D, Lanz B, Gruetter R, Cudalbu C (2023) Diffusion-weighted SPECIAL improves the detection of J-coupled metabolites at ultrahigh magnetic field. Magn Reson Med 91(1):4–18 37771277 10.1002/mrm.29805

[11] Dreher W, Busch E, Leibfritz D (2001) Changes in apparent diffusion coefficients of metabolites in rat brain after middle cerebral artery occlusion measured by proton magnetic resonance spectroscopy. Magn Reson Med 45(3):383–389 11241694 10.1002/1522-2594(200103)45:3<383::aid-mrm1050>3.0.co;2-g

[12] Choi I, Andronesi O, Barker P, Bogner W, Edden R, Kaiser L, Lee P, Marjanska M, Terpstra M, deGraaf R (2020) Spectral editing in 1H magnetic resonance spectroscopy: experts’ consensus recommendations. NMR Biomed 34(5):4411 10.1002/nbm.4411 PMC8557623 32946145

[13] Mescher M, Merkle H, Kirsch J, Garwood M, Gruetter R (1998) Simultaneous in vivo spectral editing and water suppression. NMR Biomed 11(6):266–272 9802468 10.1002/(sici)1099-1492(199810)11:6<266::aid-nbm530>3.0.co;2-j

[14] Karlicek R, Lowe I (1980) A modified pulsed gradient technique for measuring diffusion in the presence of large background gradients. J Magn Reson 37, 75-91

[15] Guo J, Gang Z, Sun Y, Laine A, Small S, Rothman D (2018) In vivo detection and automatic analysis of GABA in the mouse brain with MEGA-PRESS at 9.4 T. NMR Biomed 31(1):3837 10.1002/nbm.3837 29105210

[16] Nezhad FS, Anton A, Michou E, Jung J, Parkes LM, Williams SR (2018) Quantification of GABA, glutamate and glutamine in a single measurement at 3 T using GABA-edited MEGA-PRESS. NMR Biomed 31(1):3847 10.1002/nbm.3847 PMC5765428 29130590

[17] Minati L, Aquino D, Bruzzone M, Erbetta A (2010) Quantitation of normal metabolite concentrations in six brain regions by in vivo MR spectroscopy. J Med Phys 35:154–63 20927223 10.4103/0971-6203.62128 PMC2936185

[18] Lanz B, Abaei A, Braissant O, Choi I-Y, Cudalbu C, Henry P-G, Gruetter R, Kara F, Kantarci K, Lee P, Lutz NW, Marjańska M, Mlynárik V, Rasche V, Xin L, Valette J (2021) Magnetic resonance spectroscopy in the rodent brain: experts’ consensus recommendations. NMR Biomed 34(5) 10.1002/nbm.4325 PMC9429976 33565219

[19] Edden R, Puts N, Harris A, Barker P, Evans C (2014) Gannet: a batch-processing tool for the quantitative analysis of gamma-aminobutyric acid–edited MR spectroscopy spectra. J Magn Reson Imaging 40(6):1445–1452 25548816 10.1002/jmri.24478 PMC4280680

[20] Chen L, Weng Z, Goh L, Garland M (2002) An efficient algorithm for automatic phase correction of NMR spectra based on entropy minimization. J Magn Reson 158(1):164–168

[21] Provencher SW (1993) Estimation of metabolite concentrations from localized in vivo proton NMR spectra. Magn Reson Med 30(6):672–679 8139448 10.1002/mrm.1910300604

[22] Provencher SW (2001) Automatic quantitation of localized in vivo 1H spectra with LCModel. NMR Biomed 14(4):260–264 11410943 10.1002/nbm.698

[23] Jenkins C, Chandler M, Langbein FC, Shermer SM (2021) Benchmarking GABA quantification: a ground truth data set and comparative analysis of TARQUIN, LCModel, jMRUI and Gannet. Retrieved from arXiv:1909.02163 [physics.med-ph]

[24] Zieminska E, Toczylowska B, Diamandakis D, Hilgier W, Filipkowski R, Polowy R, Orzel J, Gorka M, Lazarewicz J (2018) Glutamate, glutamine and GABA levels in rat brain measured using MRS, HPLC and NMR methods in study of two models of autism. Front Mol Neurosci 11:418 30505268 10.3389/fnmol.2018.00418 PMC6250849

[25] Sawiak S, Jupp B, Taylor T, Caprioli D, Carpenter T, Dalley J (2016) In vivo gamma-aminobutyric acid measurement in rats with spectral editing at 4.7 T. J Magn Reson Imaging 43(6):1308–1312 26633759 10.1002/jmri.25093 PMC4869682

[26] Cudalbu C, Cavassila S, Ratiney H, Grenier D, Briguet A, Graveron-Demilly D (2006) Estimation of metabolite concentrations of healthy mouse brain by magnetic resonance spectroscopy at 7 T. Comptes Rendus Chimie 9(3–4):534–538

[27] Chen Z, Silva A, Yang J, Shen J (2005) Elevated endogenous GABA level correlates with decreased fMRI signals in the rat brain during acute inhibition of GABA transaminase. J Neurosci Res 79(3):383–391 15619231 10.1002/jnr.20364

[28] Behar K, Boehm D (1994) Measurement of GABA following GABA-transaminase inhibition by gabaculine: a H and P NMR spectroscopic study of rat brain in vivo. Magn Reson Med 31(6):660–667 7914662 10.1002/mrm.1910310612

[29] Ercan AE, Techawiboonwong A, Versluis MJ, Webb AG, Ronen I (2015) Diffusion-weighted chemical shift imaging of human brain metabolites at 7 T. Magn Reson Med 73(6):2053–2061 24986121 10.1002/mrm.25346

[30] Holz M, Heil S, Sacco A (2000) Temperature-dependent self-diffusion coefficients of water and six selected molecular liquids for calibration in accurate 1H NMR PFG measurements. Phy Chem Chem Phys 2(20):4740–4742

[31] Edden RAE, Puts NAJ, Barker PB (2012) Macromolecule-suppressed GABA-edited magnetic resonance spectroscopy at 3T. Magn Reson Med 68(3):657–661 22777748 10.1002/mrm.24391 PMC3459680

[32] Rothman DL, Petroff OA, Behar KL, Mattson RH (1993) Localized 1H NMR measurements of gamma-aminobutyric acid in human brain in vivo. Proc Natl Acad Sci 90(12):5662–5666 8516315 10.1073/pnas.90.12.5662 PMC46781

[33] Tkáč I, Deelchand D, Dreher W, Hetherington H, Kreis R, Kumaragamage C, Považan M, Spielman DM, Strasser B, de Graaf RA (2021) Water and lipid suppression techniques for advanced 1H MRS and MRSI of the human brain: experts’ consensus recommendations. NMR Biomed 34(5) 10.1002/nbm.4459 PMC8569948 33327042

[34] Ligneul C, Palombo M, Hernández-Garzón E, Carrillo-de Sauvage M-A, Flament J, Hantraye P, Brouillet E, Bonvento G, Escartin C, Valette J (2019) Diffusion-weighted magnetic resonance spectroscopy enables cell-specific monitoring of astrocyte reactivity in vivo. Neuroimage 191:457–469 30818026 10.1016/j.neuroimage.2019.02.046

[35] Döring A, Kreis R (2019) Magnetic resonance spectroscopy extended by oscillating diffusion gradients: cell-specific anomalous diffusion as a probe for tissue microstructure in human brain. Neuroimage 202:116075 31398432 10.1016/j.neuroimage.2019.116075

