# Supplementary material for: Diffusion-weighted magnetic resonance spectroscopy with selective refocusing
Source: MAGMA. 2025 Jul 15;38(6):1039–52. doi: 10.1007/s10334-025-01275-x (PMC12638348; doi:10.1007/s10334-025-01275-x)
Supplement: Supplementary file 1 — (pdf 97 KB) [file 10334_2025_1275_MOESM1_ESM.pdf]

Site	Bruker Pharmascan 70/16	Bruker BioSpin 500WB
**1. Hardware**		
Field strength [T]	7T	11.7T
Manufacturer	Bruker	Bruker
Model (software version if available)	Paravision 6.0.1	Paravision 6.0.1
RF coils: nuclei (transmit/ receive), number of channels, type, body part	55mm/23mm tx/rx quad head coil	MICWB40 RES 500 1H 010 / 10 LTR
Additional hardware		
**2. Acquisition**		
Pulse sequence		
Volume of Interest (VOI) locations	central brain region	center of 10mm tube
Nominal VOI size [mm^3]	196	64
Repetition Time (TR), Echo Time (TE) [ms, s]	TE=68ms TR=2.5s	TE=68ms TR=2.5s
Total number of Excitations or acquisitions per spectrum	32	16
Number of Averaged spectra (NA) per time-point	32	16
Averaging method (e.g. block-wise or moving average)	block-wise	block-wise
Total number of spectra (acquired / in time-series)	32	32
Additional sequence parameters (spectral width in Hz, number of spectral points, frequency offsets)	SW= 3278Hz, 4096 points	SW=5500Hz, 4096 points
Water Suppression Method	VAPOR	VAPOR
Shimming Method, reference peak, and thresholds for "acceptance of shim" chosen	Automated 3D B_0 field mapping technique, followed by localized shim before each scan $< 12Hz$	Automated 3D B_0 field mapping technique, followed by localized shim before each scan $< 3Hz$
Triggering or motion correction method		
**3. Data Analysis Methods and Outputs**		
Analysis software	Spectra were processed by an in-house developed MATLAB pipeline based on the established GANNET pipeline. Signals were phased to the region of interest in the spectrum.	Spectra were processed by an in-house developed MATLAB pipeline based on the established GANNET pipeline. Signals were phased to the region of interest in the spectrum.
Processing steps deviating from quoted reference or product	ACME phase correction algorithm	ACME phase correction algorithm
Output measure (e.g. absolute concentration, institutional units, ratio)	Intensity in arbitrary units, attenuation, b-values.	Intensity in arbitrary units, attenuation, b-values.

Quantification references and assumptions, fitting model assumptions	Acquired spectra are in same units, I.E 100 in one spectra is equal to 100 in the next	Acquired spectra are in same units, I.E 100 in one spectra is equal to 100 in the next
**4. Data Quality**		
Reported variables (SNR, Linewidth)	Calculated ADC, SNR of the peak of interest	Calculated ADC, SNR of the peak of interest
Data exclusion criteria	Failed water suppression, water signal > 20 times NAA signal intensity	NA
Quality measures of post-processing Model fitting (e.g. CRLB, goodness of fit, SD of residual)	Fit error calculated using: $\epsilon_{metab} = 100 \cdot \frac{std(resid_{metab})}{A_{metab}}$ according to the GANNET pipeline, where $resid_{metab}$ is signal model fit residuals and A_{metab} is signal model amplitude	Fit error calculated using: $\epsilon_{metab} = 100 \cdot \frac{std(resid_{metab})}{A_{metab}}$ according to the GANNET pipeline, where $resid_{metab}$ is signal model fit residuals and A_{metab} is signal model amplitude
