# Supplementary material for: Diffusion-weighted magnetic resonance spectroscopy with selective refocusing
Source: MAGMA. 2025 Jul 15;38(6):1039–52. doi: 10.1007/s10334-025-01275-x (PMC12638348; doi:10.1007/s10334-025-01275-x)
Supplement: Supplementary file 2 — (pdf 210 KB) [file 10334_2025_1275_MOESM2_ESM.pdf]

13-interval PGSE Calculation of b-value

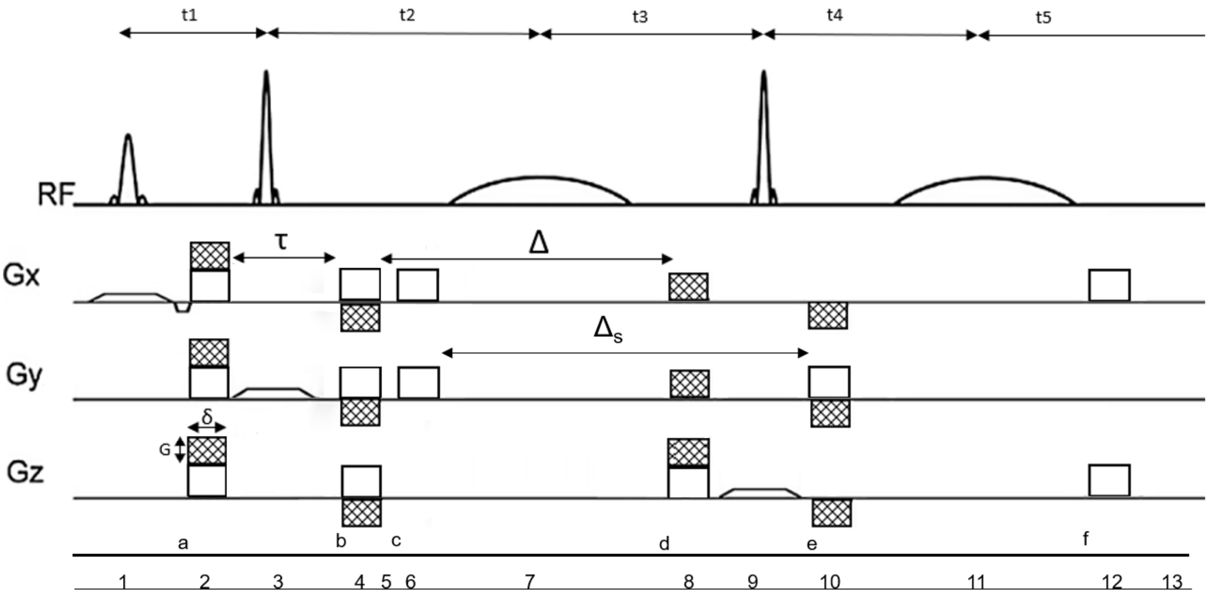

Figure 1: Caption describing the figure.

The b-value is calculated using the 13-interval PGSE:

$$b = \sum_{i=1}^{13} \int_{t_{i-1}}^{t_i} \left(\int_{t'_{i-1}}^{t'_i} (g(t'') + s(t''))\, dt'' \right)^2 \tag{1}$$

For the timings, the 13 intervals are:

$$\begin{array}{rlll rlll}
1: & 0 & \to a & & 8: & d & \to d+\delta \\
2: & a & \to a+\delta & & 9: & d+\delta & \to e \\
3: & a+\delta & \to b & & 10: & e & \to e+\delta \\
4: & b & \to b+\delta & & 11: & e+\delta & \to f \\
5: & b+\delta & \to c & & 12: & f & \to f+\delta \\
6: & c & \to c+\delta & & 13: & f+\delta & \to \text{end} \\
7: & c+\delta & \to d & & & &
\end{array}$$

With the gradient strengths (for the z-direction):

$$\begin{array}{rl rl}
1: & G_0 & 8: & G_0 - G - S \\
2: & G_0 + G + S & 9: & G_0 \\
3: & G_0 & 10: & G_0 - G \\
4: & G_0 + G - S & 11: & G_0 \\
5: & G_0 & 12: & G_0 + S \\
6: & G_0 & 13: & G_0 \\
7: & G_0 & &
\end{array}$$

The term G_0 is included to represent a constant background gradient, for this system however, we assume it is 0. Doing this calculation will end up with:

$$\delta^2 G^2(3d - a - 3b + e - \frac{2}{3}\delta) + \delta^2 S^2(-a + b - c + f - \frac{2}{3}\delta) + \delta^2 GS(4c - 2d - 2e - 2a + 2b) \tag{2}$$

From here we separate out the main diffusion part, the mixing term, and the constant attenuation caused by the spoilers. We also convert to Δ and τ form by using the definitions from the sequence diagram, in this case:

$$\Delta = d - (b + \delta) \tag{3}$$

$$\tau = b - (a + \delta) = e - (d + \delta) \tag{4}$$

$$\Delta_S = e - (c + \delta) \tag{5}$$

Main diffusion term:

$$\delta^2 G^2(3(d - b) + (e - a) - \frac{2}{3}\delta) = (2\delta)^2 G^2(\Delta + \frac{1}{2}\tau + \frac{4}{3}\delta) \tag{6}$$

Mixing terms:

$$\text{(x-component)} \quad \delta^2 G_x S_x(4c - 2d - 2e - 2a + 2b) = -(2\delta)^2 G_x S_x(\Delta_S - \tau) \tag{7}$$

$$\text{(y-component)} \quad \delta^2 G_y S_y \left(4c - 2d - 2e - 2a + 2b + \frac{\delta}{3}\right) = -(2\delta)^2 G_y S_y \left(\Delta_S - \tau - \frac{\delta}{12}\right) \tag{8}$$

$$\text{(z-component)} \quad \delta^2 G_z S_z \left(2d + 2b - 2e - 2a - \frac{\delta}{3}\right) = -(2\delta)^2 G_z S_z \left(\frac{\delta}{12}\right) \tag{9}$$
