# Supplementary figures and images for: Diffusion-weighted magnetic resonance spectroscopy with selective refocusing
Source: MAGMA. 2025 Jul 15;38(6):1039–52. doi: 10.1007/s10334-025-01275-x (PMC12638348; doi:10.1007/s10334-025-01275-x)

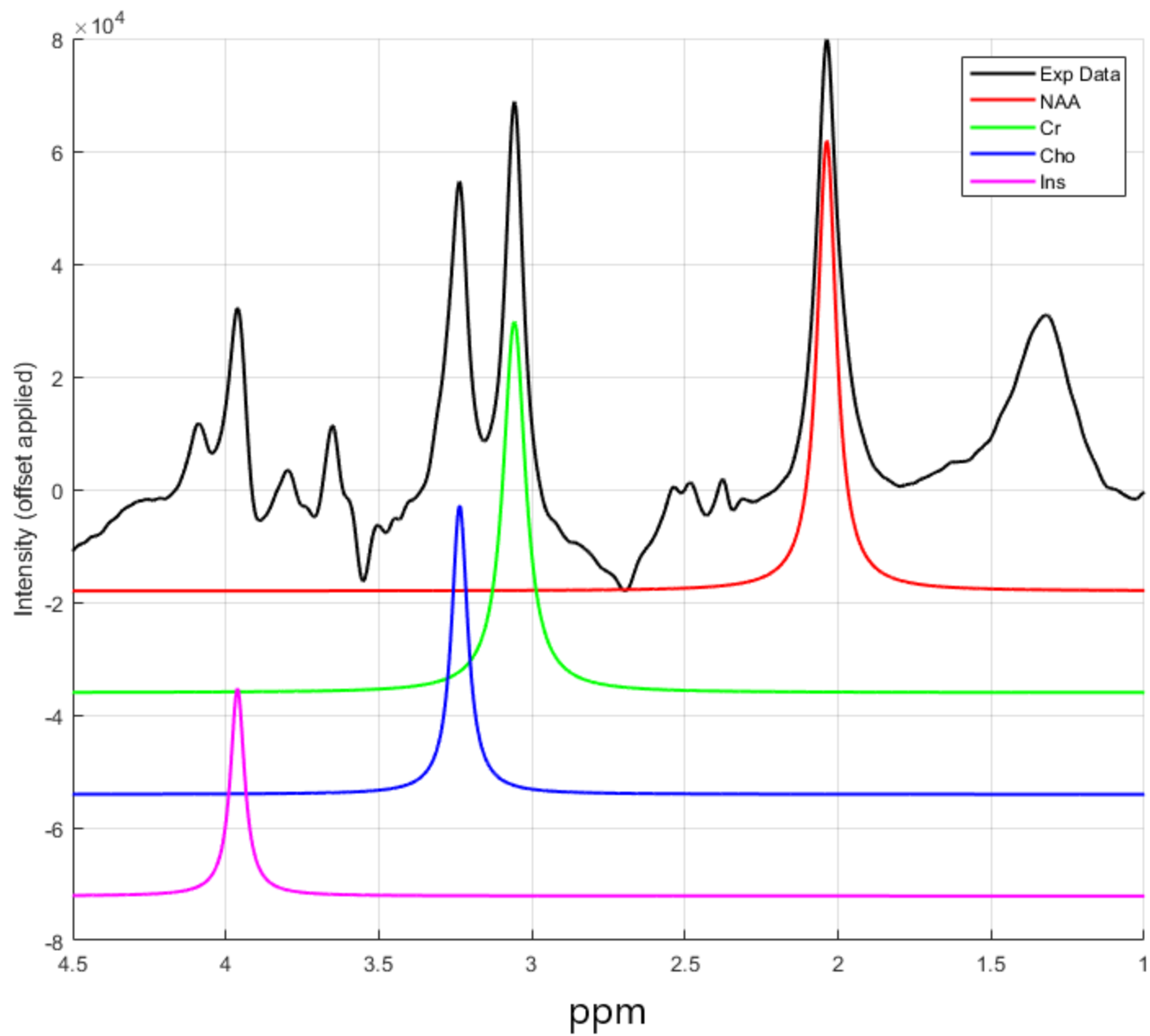

Supplement: Supplementary file 3 — (pdf 53 KB) [file 10334_2025_1275_MOESM3_ESM.pdf]

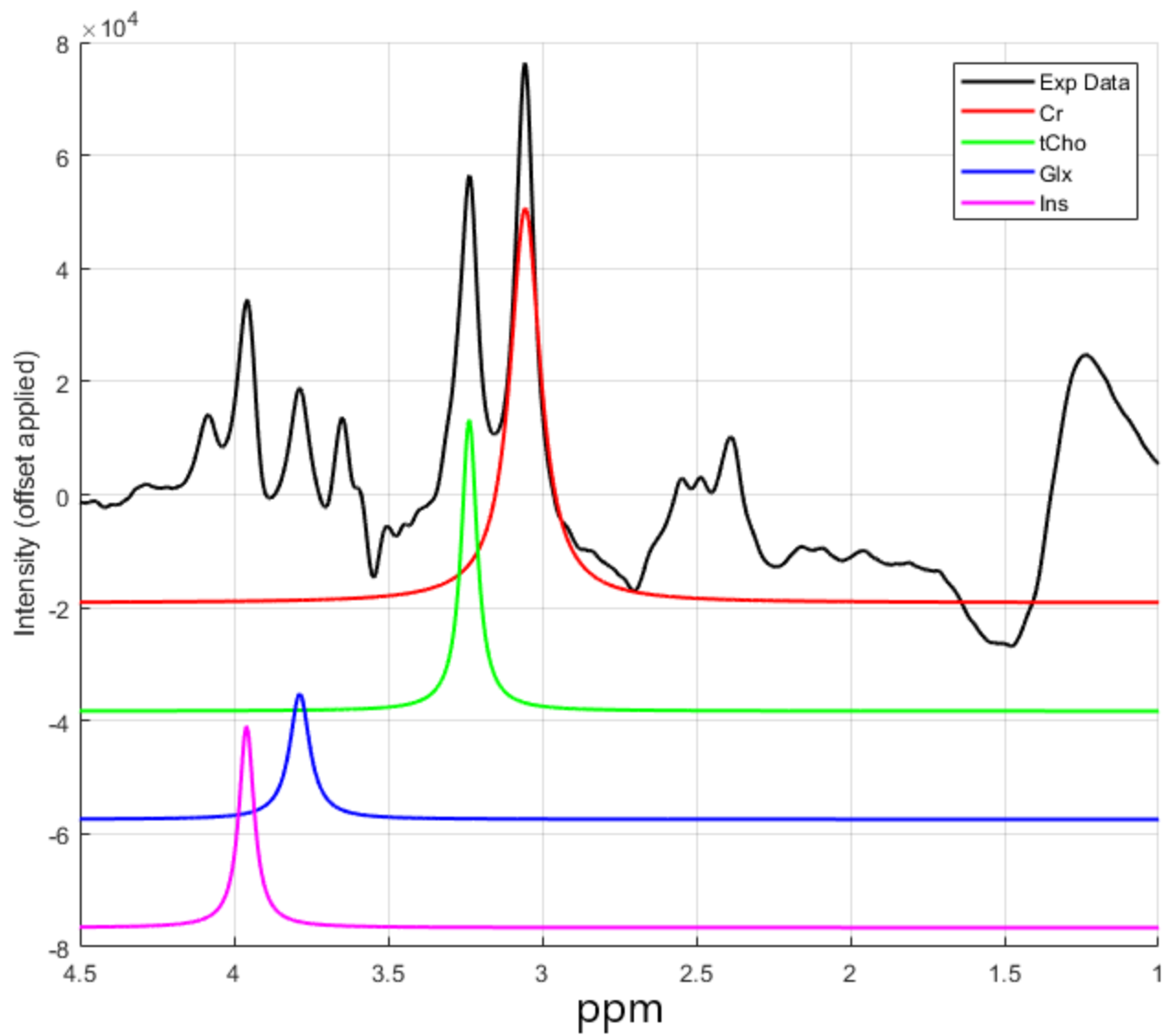

Supplement: Supplementary file 4 — (pdf 45 KB) [file 10334_2025_1275_MOESM4_ESM.pdf]
